# Paired Measurements of Paraoxonase 1 and Serum Amyloid A as Useful Disease Markers

**DOI:** 10.1155/2013/481437

**Published:** 2013-10-22

**Authors:** Kazuhiko Kotani, Toshiyuki Yamada, Alejandro Gugliucci

**Affiliations:** ^1^Department of Clinical Laboratory Medicine, Jichi Medical University, Shimotsuke-shi, Tochigi 329-0498, Japan; ^2^Glycation, Oxidation and Disease Laboratory, Touro University-California, Vallejo, CA 94592, USA

## Abstract

Paraoxonase 1 (PON1) and serum amyloid A (SAA) are proteins carried by high-density lipoprotein (HDL) particles. Among the HDL-associated protein molecules, SAA, an inflammation-related marker, and PON1, an antioxidant marker, tend to change in relatively clear opposite directions in physiological situations. In clinical chemistry, paired measurements of both markers may provide useful information to understand dysfunctional HDL in diseases with inflammation and oxidative stress conditions. Actually, limited clinical studies have suggested that the combined use of PON1 and SAA may be a tool for observing the pathophysiology of some disease entities. From the findings of experimental studies, PON1 appears to be cooperatively regulated by inflammation- and oxidative stress-related molecules linked with SAA regulation in humans. More studies remain to be performed to ascertain the value of paired measurements of both promising markers in clinical practice.

## 1. Introduction

High-density lipoprotein (HDL) directs cholesterol efflux and reverse-cholesterol transport from peripheral tissue to the liver, with multiple vital functions that include antioxidant, anti-inflammatory, antiapoptotic, nitric oxide-promoting, immunomodulating, and antithrombotic effects [1–4]. Epidemiological and clinical studies have shown that low circulating HDL-cholesterol levels are associated with an increase of cardiometabolic risk [5, 6]. The HDL biology is very complex but is currently at the frontier in atherosclerotic research with a huge potential for positive public health implications.

With the progression of our understanding of HDL particles, multiple protein molecules that coisolate with HDL have attracted great attention [7]. The small dense HDL subclass (as HDL3c) is a fraction associated with the development of cardiometabolic diseases [8]. The subclass preferentially and sometimes exclusively carries several proteins and/or displays array of proteins [7, 9]. Among these molecules, for instance, paraoxonase 1 (PON1) and serum amyloid A (SAA), respectively, have been the objects of multiple studies. PON1, an HDL-associated apolipoprotein, can exert some of its physiological functions by eliminating oxidative molecules and lactones and/or by hydrolyzing them to nontoxic moieties [10, 11]. A role of PON1 has been revealed in a wide range of pathologic conditions [12–15]. SAA, another HDL-associated apolipoprotein, is well known as an acute-phase protein during inflammation [16–18]. A causal relevance of SAA has been shown not only in inflammatory diseases but also in cardiometabolic diseases [12, 16–19].

**Figure 1:**
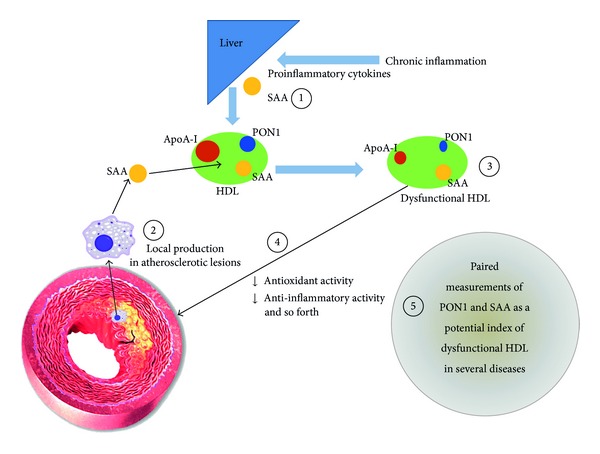
A speculative interplay between PON1 and SAA in HDL particles. Apo-I: apolipoprotein A-I, HDL: high-density lipoprotein, PON1: paraoxonase 1, and SAA: serum amyloid A. Based on well-accepted evidence, chronic inflammation induces the secretion of SAA by the liver via cytokine signaling as depicted in (1). SAA may also stem from local extrahepatic synthesis at the site of atherosclerotic lesions (2). Under such conditions of increased SAA, a reduction of PON1 activity and apoA-I is seen in HDL particles. This renders a functionally deficient HDL particle (dysfunctional HDL) (3), for instance, which has less anti-inflammatory and antioxidant effects (4). Paired measurements of both SAA and PON1 may offer useful information on these pathways of dysfunctional HDL in several disease entities and deserve future basic and clinical studies as a potential biomarker pair (5).

Inflammation and oxidative stress are coexisting conditions underlying chronic diseases [20–22], and under such conditions, *nascent *native HDL particles can be converted into a more proatherogenic form of HDL particles [23, 24] with an altered complement of HDL-associated proteins, that is, an increase in SAA and a decrease in PON1. Because SAA, an inflammation-related marker, and PON1, an antioxidant marker, have relatively clear opposite characteristics among the HDL-associated proteins, the approach of assessing the two markers simultaneously may provide new insights in clinical practice (Figure 1).

## 2. Clinical Studies on PON1 and SAA

Despite the suggested role of the two markers in chronic diseases, a few studies that investigated both PON1 and SAA levels exist in the clinical setting [12, 19]. The first study examined these markers in patients with rheumatoid arthritis (*n* = 64, 5 men and 59 women, mean age 63 years) [12]. The patients with rheumatoid arthritis showed a significantly lower serum PON1 (paraoxon) activity (mean 131 *μ*mol/min/L) than that of healthy control subjects (164 *μ*mol/min/L). On the other hand, the SAA level was significantly higher in those with rheumatoid arthritis (mean 63 mg/L) than in healthy control subjects (mean 9 mg/L). The ratio of PON1 to SAA was lower in patients with rheumatoid arthritis than in healthy control subjects. There was a nonsignificant but mild inverse correlation between PON1 and SAA (coefficient = −0.10).

The increase of SAA was followed over time in patients with rheumatoid arthritis [12]. The authors also indicated several possible mechanisms for the reduction of PON1 level in rheumatoid arthritis: consumption/inactivation of antioxidant PON1 property under the disease with inflammation and oxidative stress, the influence of structural/compositional and functional changes of HDL particles modified by inflammation and oxidative stress on the active sites of PON1, a rapid clearance of PON1 during inflammation, and/or a disturbance of PON1 production by the liver at the transcriptional level during inflammation [12]. The study might thus imply the potential usefulness of measuring such inflammation-related and antioxidant markers simultaneously for better understanding of the pathophysiology of diseases and their severity or monitoring clinical course.

Another study examined the correlation between PON1 and SAA levels in nondiabetic patients (*n* = 86, 46 men and 40 women, mean age 55 years) [19]. The patients with metabolic syndrome showed a significantly lower level of PON1 (arylesterase) activity (mean 111 AU) than those without metabolic syndrome (mean 133 AU). By contrast, the patients with metabolic syndrome showed a significantly higher level of SAA (median 1.61 mg/L) than those without metabolic syndrome (median 1.10 mg/L). Simple and multiple linear regression analyses for all patients revealed that the PON1 activity level was significantly inversely correlated to the SAA level (*r* = −0.28, *β* = −0.26 to −0.29). The authors speculated that dysfunctional HDL might ensue in part due to the increase of SAA via the PON1 regulation [19].

## 3. Experimental Studies on PON1 and SAA

There have been several experimental studies that investigated the relationship between PON1 and SAA [25–28]. Most studies have focused on how SAA regulates PON1 metabolism.

The first study investigated HDL particles during an acute-phase response in a croton oil rabbit model and revealed that SAA in HDL particles was elevated, and, concomitantly, PON1 (arylesterase) activity in HDL was reduced [25]. From the data, the hypothesis was posited that the reduction of PON1 might be due to inflammatory molecules, such as SAA [25]. Another explanation might be the exchange of apolipoprotein A-I (apoA-I) for SAA, with consequent decrease in PON1 activation that requires apoA-I for optimal catalysis.

Another group was set out to assess PON1 regulation by SAA as well as the other inflammation-related molecules that can induce an increase in SAA [26]. That study revealed that PON1 mRNA expression in human hepatoma HepG2 cells was reduced following the stimulation with, not SAA, but inflammatory cytokines, such as interleukin-1*β* (IL-1*β*) and tumor necrosis factor-*α* (TNF-*α*) [26]. The other study compared the fractional change of distribution of PON1 (arylesterase) activity by SAA among the sera of humans, rabbits, and mice [27]. That study revealed that the response of PON1 to SAA was somewhat different among the three species; that is, following incubation with SAA, PON1 was displaced from mice HDL in particular and to a lesser extent from rabbit or human HDL [27].

Although an additional study did not demonstrate PON1 regulation by direct exposure to SAA [28], that study revealed that SAA mRNA expression was elevated, while in a coordination and reciprocal manner, PON-1 mRNA expression was reduced in murine hepatoma Hepa 1-6 and hepatocyte AML12/NMH cells following the simultaneous stimulation with TNF-*α*, IL-1*β*, and IL-6. Subsequently, animal studies revealed the SAA expression by nuclear factor-kappa B, NF-*κ*B, transactivation, while showing PON-1 expression by inhibiting peroxisome proliferator-activated receptor-*α*, PPAR*α*, activation [28].

## 4. Perspectives

In the clinical setting, the paired measurements of PON1 and SAA may be a tool to gain insight on the dysfunctional HDL status in some disease entities and disease severity or to monitor the therapeutic effects. Available previous clinical studies [12, 19] are pioneer works that suggest potential usefulness for the concurrent measurement of PON1 and SAA; however, unfortunately the relationship between PON1 and SAA was not fully studied in terms of disease severity and pharmaceutical treatment in these studies. The assessment of HDL functions, such as cell culture-based assays used in general [29], was not performed in these studies, although one study [12] showed a low concentration of serum lecithin-cholesterol acyltransferase (LCAT: seemingly one of the markers related to HDL functions) in patients with rheumatoid arthritis and the PON1 level to correlate with the LCAT level in these patients.

Which diseases would benefit from the combined use of the two markers in clinical practice remain to be determined. How relevant to dysfunctional HDL is this approach should be further investigated as well. Dysfunctional HDL has been suggested to contribute to the development of atherosclerotic and/or cardiometabolic diseases [7–9]; therefore, for instance, clinical studies using the paired measurements of PON1 and SAA could be envisaged in cohort studies designed to observe cardiometabolic outcomes. Whereas studies of HDL functions require complex and expensive assays using cell culture, PON1 and SAA assays have the advantage of being easily performed in the clinical laboratories. Studies designed to gather further evidence of the usefulness of paired measurements of PON1 and SAA would offer an interesting avenue for the development in clinical pathology.

From the experimental findings reviewed regarding a direct influence of SAA on PON1 regulation in humans, PON1 appears to be cooperatively regulated by inflammation- and oxidative stress-related molecules linked with SAA regulation. For basic understanding of the paired measurements of PON1 and SAA, the PON1-SAA relationship deserves to be more thoroughly investigated in experimental studies.

SAA and PON1 are chiefly produced by the liver, while localized synthesis of SAA has been shown in atherosclerotic lesions, which are accompanied by inflammation and oxidative stress [18]. PON1 is also seen in atherosclerotic lesions [30], and a key function of PON1 is to control macrophage oxidation and foam cell formation [31]. We may advance the hypothesis that the extrahepatic production of SAA can promote the dissociation of PON1 from HDL particles, most notably in the site of atherosclerotic lesions. However, whether such an extrahepatic phenomenon occurs in the presence of relatively low SAA levels should be carefully addressed.

## 5. Conclusions

Both PON1 and SAA are proteins carried by HDL particles with physiologically divergent characteristics. Measuring both markers simultaneously can be envisaged as an index that may play the role of a surrogate marker of dysfunctional HDL in inflammation and oxidative stress conditions. The combined use of PON1 and SAA may turn out to be a useful tool in clinical practice. Further clinical and experimental studies are therefore warranted.
